# Soufeng sanjie formula alleviates bone erosion in CIA mice via inhibiting RANKL/NF-κB signaling pathway and ameliorates the RA symptom in patients

**DOI:** 10.3389/fphar.2025.1604180

**Published:** 2025-07-31

**Authors:** Dan Lin, Yutong Wu, Lizhong Zhu, Jie Yang, Jianbin Ge, Xiaoyan Sun, Xueting Cai, Juan Ye, Zhonghua Pang, Jiao Chen, Chunping Hu

**Affiliations:** ^1^Jiangsu Provincial Medical Innovation Center, Affiliated Hospital of Integrated Traditional Chinese and Western Medicine, Nanjing University of Chinese Medicine, Nanjing, Jiangsu, China; ^2^ College of Clinical Chinese Medicine, Gansu University of Chinese Medicine, Lanzhou, Gansu, China; ^3^Department of Pharmacy, The Second People’s Hospital of Nantong, Nantong, Jiangsu, China

**Keywords:** Soufeng sanjie formula, rheumatoid arthritis, bone erosion, osteoclast, RANKL/NF-κB

## Abstract

**Aim of the study:**

Soufeng sanjie formula (SF), composed by scolopendra, scorpion, astragali radix and black soybean seed coats, is an in-hospital preparation of traditional Chinese medicine used for the treatment of rheumatoid arthritis (RA). It has been demonstrated to have a prominent effect on relieving the symptoms of collagen-induced arthritis (CIA) mice after 1 month of administration. This study aimed to evaluate the effect and mechanism of SF on ameliorating bone erosion in CIA mice and RA patients.

**Materials and Methods:**

SF or methotrexate (MTX) was administered orally to CIA mice for 3 months. The degree of ankle joint destruction, osteoclast counts, bone erosion and the expression of osteoclast-related proteins were evaluated in the ankle joints of CIA mice. Then, the inhibitory effect of SF on RANKL-stimulated osteoclast differentiation was investigated in bone marrow-derived mononuclear cells *in vitro*, with a focus on NF-κB signaling activation. Additionally, a preliminary clinical study was conducted to evaluate the effect of SF monitoring the serum levels of bone erosion-related factors (IL-6, IL-10, OPG and TRACP) and liver and kidney functions were monitored in RA patients.

**Results:**

SF significantly relieved the symptoms of arthritis and ameliorated bone erosion in CIA mice after 3 months of treatment, without obvious toxicity in normal ICR mice. In addition, it decreased the number of osteoclasts and reduced the expression of cathepsin K in the ankle joints of CIA mice. *In vitro* experiments demonstrated that SF inhibited RANKL-induced osteoclast differentiation and reduced cathepsin K and MMP9 expression in bone marrow-derived mononuclear cells. Furthermore, SF reduced p65 phosphorylation and blocked p65 nuclear translocation. Clinical observation confirmed that SF effectively improved the clinical symptoms of RA patients and regulated the serum levels of bone erosion-related factors (IL-6, IL-10, OPG and TRACP), without obvious toxicity to the patient’s liver or kidneys.

**Conclusion:**

This study confirmed that SF effectively relieves arthritis symptoms in both CIA mice and RA patients without causing obvious toxicity to the liver or kidney. The therapeutic effects were mediated through amelioration of bone erosion by inhibiting the RANKL/NF-κB signaling pathway-mediated osteoclast differentiation.

## Introduction

Rheumatoid arthritis (RA) is an autoimmune disease that characterized by progressive joint and damage and a high risk of disability (Das and Padhan, 2017). It affects approximately 0.5%–1% of the global population, with peak incidence observed in individuals aged 30–50 years (Finckh et al., 2022). As a progressive disease, bone erosion is an important pathological hallmark and occurs throughout RA. This irreversible damage to cartilage, bone and other adjacent tissues ultimately leads to severe joint deformity and even disability, imposing great psychological and economic burdens to RA patients and their families while substantially compromising quality of life (Maeda et al., 2022). Current therapeutic strategies, including disease-modifying anti-rheumatic drugs (DMARDs) and nonsteroidal anti-inflammatory drugs (NSAIDs), primarily target inflammatory symptoms but fail to prevent the progression of bone erosion (Das and Padhan, 2017; Figus et al., 2021). Therefore, identifying therapeutics that simultaneously alleviate symptoms, inhibit bone erosion, and minimize adverse effects represents a critical unmet need in RA treatment.

The pathogenesis of RA-associated bone erosion stems from an imbalance in bone remodeling, characterized by excessive osteoclast-mediated bone resorption coupled with impaired osteoblast activity (Jung et al., 2014; Ridgley et al., 2018). This dysregulation is primarily governed by the receptor activator of nuclear factor κB (RANK), RANK ligand (RANKL) and osteoprotegerin (OPG)-pivotal regulators of osteoclast formation, activation, development, and maturation (Ando et al., 2008; Gao et al., 2021; Takegahara et al., 2022). Osteoclast differentiation relies on the RANKL/RANK-mediated NF-κB signaling pathway (Fang et al., 2020). In the inflammatory microenvironment, RANKL binding activates the NF-кB signaling pathway, leading to IκB kinase (IKK) activation. IKK promotes the phosphorylation of IκB, which is subsequently ubiquitinated and degraded by ubiquitin ligase. Then, NF-кB dissociates to form free p65, which initiates the transcription of osteoclast-related genes after translocates to the nucleus (Jimi et al., 2019; Yao et al., 2022). RANKL-RANK interaction triggers intracellular signaling pathways that ultimately initiate osteoclast differentiation and the expression of specific genes, such as cathepsin K, matrix metalloproteinase 9 (MMP9) and tartrate-resistant acid phosphatase (TRACP) (Borggaard et al., 2020; Ali et al., 2021; Han et al., 2021; Shin et al., 2021; Remmers et al., 2023).

The traditional Chinese medicine Soufeng Sanjie formula (SF), composed of Astragali Radix (dried root of *Astragalus membranaceus* (Fisch.) Bge), scorpion (dried body of *Buthus martensii* Karsch), scolopendra (dried body of *Scolopendra subspinipes mutilans* L. Koch), and black soybean seed coats (seed coats of *Glycine max* (L.) Merr), is an in-hospital preparation used for the treatment of RA, which has been used clinically for many years and the curative effect is remarkable. Experimental study has demonstrated that SF significantly alleviates paw swelling, decreases arthritis scores and reduces cartilage loss in CIA mice after 1 month of administration (Hua et al., 2021). Since treating bone erosion requires long-term intervention, we administered SF to CIA mice for 3 months in the present study to evaluate its effects on bone erosion improvement and preliminarily investigate the possible mechanism involved. Furthermore, we clinically validated the therapeutic efficacy of SF in RA patients.

## Materials and methods

### Animals

Female DBA/1J, C57BL/6 and ICR mice were purchased from Changzhou Cavens Experimental Animal Co., Ltd. (Jiangsu, China). All mice were raised at the Animal Center of Jiangsu Province Academy of Traditional Chinese Medicine. This study was performed according to the Animal Welfare Law of China and approved by the Animal Ethics Committee of Jiangsu Province Academy of Traditional Chinese Medicine (AEWC-20200819-125).

### Reagents

Methotrexate (MTX, Shanghai Xinyi Pharmaceutical Co., Ltd.) and processed scorpions, scolopendra, astragali radix, and black soybean seed coats were purchased from the Affiliated Hospital of Integrated Traditional Chinese and Western Medicine, Nanjing University of Chinese Medicine (Nanjing, Jiangsu, China). Freund’s adjuvant and bovine type II collagen were obtained from Chondrex (Redmond, WA, United States). Tartrate-resistant acid phosphatase (TRAP) staining kits were obtained from Sigma‒Aldrich (St. Louis, MO, United States). M-CSF was purchased from Pepro Technology (Rocky Hill, NJ, United States). IL-6, RANKL, OPG and Cathepsin K antibodies were purchased from AiFang Biological Company (Changsha, Hunan, China). Antibodies against phosphorylated p65, p65 and MMP9 were obtained from Cell Signaling Technology (Danvers, MA, United States).

### Preparation of SF

The SF formula consists of 20 g Astragali Radix (dried root of *A. membranaceus* (Fisch.) Bge), 0.5 g scorpion (dried body of *B. martensii* Karsch), 0.5 g scolopendra (dried body of *Scolopendra subspinipes mutilans* L. Koch), and 30 g black soybean seed coats (seed coats of *G. max* (L.) Merr). The SF used in both animal and cell experiments was provided by the pharmacy of Jiangsu Province Hospital on Integration of Chinese and Western Medicine (Jiangsu, China) and prepared according to our previously published methods (Hua et al., 2021). Specifically, the animal components (scorpion and scolopendra) were ground through an 80-mesh sieve and further pulverized using a cryogenic ball mill. The botanical components (astragali radix and black soybean seed coats) were decocted in water, followed by freeze-drying at −80 C to obtain powder. In in vitro and *in vivo* experiments, the combined powders were dissolved in sterile water for use. The SF used for clinical administration in RA patients was manufactured by Jiangyin Tianjiang Pharmaceutical Co., Ltd., batch No. 2105317. The formulation and preparation method of SF for *in vitro* and *in vivo* experiments was consistent with that of the clinically used SF manufactured by Jiangyin Tianjiang Pharmaceutical Co., Ltd. Moreover, the SF batch used in the pharmacological and mechanistic studies was the same as that used in our recent article (Fan et al., 2025), thus the chemical profile characterized by UHPLC-QE-MS in that article applies directly here.

### Assessment of the toxicity of SF in ICR mice

ICR mice were randomized divided into a normal control group and an SF-treated group (n = 5). Based on clinically equivalent human doses and our previous publication (Hua et al., 2021), SF was administered orally at the dose of 183 mg/kg/day for 3 months. The mice in the normal group were given an equal volume of double distilled water. After 3 months of continuous treatment, blood samples were collected via retro-orbital bleeding prior to euthanasia by cervical dislocation. Potential toxicity of SF was assessed by detecting serum biochemical indices.

### CIA mouse model induction and animal management

The CIA model was established according to our previously published protocol, with an approximate success rate of 80% (Hua et al., 2021; Ling et al., 2021). The female DBA/1J mice were randomly divided into five groups (n = 6): the normal control group (Normal), CIA vehicle group (CIA Vehicle), MTX group (920 mg/kg), high-dose SF group (SF-H, 550 mg/kg) and low-dose SF group (SF-L, 183 mg/kg). The dosing regimens for MTX and SF were determined according to the clinically recommended doses for humans in reference to our previous publication (Hua et al., 2021). Drug administration was initiated 28 days after the first immunization with bovine type II collagen. SF was administered orally at 183 or 550 mg/kg/day, and MTX was administered at 920 mg/kg twice a week. Both normal and model control groups received equivalent volumes of double distilled water.

### Arthritis assessment

Mice were monitored for arthritis progression through regular assessments of arthritis scores, paw swelling, and body weight every 3–4 days after the first immunization. Arthritis severity was scored on a 0–4 scale for each limb according to established criteria (Hua et al., 2021), with the cumulative score of all four limbs (maximum 16) representing the individual mouse’s total arthritis score (Filippin et al., 2013). The swelling was evaluated by a paw swelling meter (Woodland Hills, CA, United States).

### Histopathological evaluation and micro-CT scan

The mice were sacrificed by neck dislocation 90 days after treatment. The hind limb tissue sections were processed and stained with hematoxylin-eosin (H&E). The degree of histopathological damage was scored according to our previously described criteria (Ling et al., 2021). The hind limbs were scanned with a compact micro-computed tomography (micro-CT) scanner (Bruck SkyScan 1176, Germany). The bone mineral density (BMD) and bone volume fraction (BV/TV), which reflect bone mass, were evaluated with the built-in software.

### Osteoclast identification and immunohistochemistry

Osteoclasts in the ankle joint sections of the mice were identified by TRAP staining. IL-6, RANKL, OPG and cathepsin K were immunologically localized by the respective primary antibodies. The images were processed by optical microscopy and analyze dby Aipathwell (Dogan et al., 2019; Guo et al., 2019; Paschalis et al., 2019; Maclean et al., 2020).

### Cell culture

Bone marrow-derived mononuclear cells from the tibia and femur were isolated from 6-8-week-old C57BL/6 mice. The cells were cultured in α-MEM supplemented with 10% fetal bovine serum, 1% penicillin/streptomycin, and 30 ng/mL M-CSF at 37°C with 5% CO_2_ for 3 days. The adherent cells left at the bottom of the culture dish were considered bone marrow-derived mononuclear cells.

### Cell viability assay

SF was filter-sterilized through a 0.22 μm PVDF filter membrane for *in vitro* experiments. Cell viability was assessed using a Cell Counting Kit-8 (CCK-8) assay. Bone marrow-derived mononuclear cells were cultured in medium containing 30 ng/ml M-CSF. After adhesion, cells were treated with different concentrations of SF (0.01–20 mg/mL) for 72 h. Absorbance values were measured 2 h after the addition of CCK-8 reagent.

### Osteoclast differentiation and TRAP staining *in vitro*

Bone marrow-derived mononuclear cells were pretreated with or without SF (0.1, 0.5, or 1 mg/mL) for 2 h and then stimulated with RANKL (100 ng/mL) for 5 days, after which the solution was changed every 2 days. On the fifth day of culture, osteoclasts were stained using a TRAP staining kit and observed with an optical microscope.

### RNA extraction and RT‒qPCR analysis

Bone marrow-derived mononuclear cells were pretreated with or without SF for 2 h and then stimulated with RANKL for 48 h. Total RNA was subsequently extracted and reverse-transcribed to cDNA. RT-qPCR analysis was performed using SYBR Green qPCR Master Mix with cDNA as a template. Relative gene expression was calculated using the ^2−ΔΔCT^ method. The primer sequences are presented in Table 1.

**TABLE 1:** Primers used for RT‒qPCR.

Gene	Forward	Reverse
Murine MMP9	CTG​GAC​AGC​CAG​ACA​CTA​AAG	CTC​GCG​GCA​AGT​CTT​CAG​AG
Murine cathepsin K	GAA​GAA​GAC​TCA​CCA​GAA​GCA​G	TCC​AGG​TTA​TGG​GCA​GAG​ATT
Murine β-actin	AAC​AGT​CCG​CCT​AGA​AGC​AC	CGT​TGA​CAT​CCG​TAA​AGA​CC

### Western blotting and immunofluorescence

The expression levels of cathepsin K, p65 and phosphorylated p65 (p-p65) in bone marrow-derived mononuclear cells were detected by Western blotting. Analyses were performed using ImageJ software. In addition, bone marrow-derived mononuclear cells were treated with or without SF for 12 h and then stimulated with RANKL for 1 h. The nucleation of p65 was detected by immunofluorescence staining.

All antibodies and reagents used for Western blotting are shown in the Table 2.

**TABLE 2:** Antibodies Information for Western blottin**g**.

Target	Company	Host species	Dilution	Blocking buffer
Cathepsin K	AiFang biological company	Mouse	1:1000	5% BSA
p65	Cell signaling technology	Mouse	1:1000	5% BSA
p-p65	Cell signaling technology	Mouse	1:1000	5% BSA
GAPDH	Cell signaling technology	Mouse	1:1000	5% BSA
Secondary antibody	Cell signaling technology	Rabbit anti-mouse	1:10,000	5% BSA

### Clinical observation

The study followed the principles of the Declaration of Helsinki for humans and was approved by the ethics committee of the Affiliated Hospital of Integrated Traditional Chinese and Western Medicine, Nanjing University of Chinese Medicine (2022-LWKY-038). Informed consent was obtained, and the study was registered at the Chinese Clinical Trials Registry (ChiCTR2200066446). All patients in the study met the American College of Rheumatology/European League Against Rheumatism-2010 (ACR/EULAR-2010) diagnostic criteria for RA. The inclusion criteria for the participants were as follows: age between 18 and 70 years; no use of slow-acting anti-rheumatic drugs or use of slow-acting anti-rheumatic drugs with a stable dosage for more than 6 weeks; no use of NSAIDS/glucocorticoids or use of NSAIDS/glucocorticoids with a stable dosage for more than 2 weeks. Exclusion criteria included: pregnancy, hepatic/renal dysfunction, hypertension (grade ≥2), significant cardiovascular/cerebrovascular diseases, malignancies, or other serious medical conditions.

Following the eligibility assessment, 16 RA patients were enrolled in this study and randomly divided into an MTX group and an SF + MTX group at a 1:1 ratio. Patients in both groups were scheduled to receive MTX tablets at an initial dosage of 10 mg/week, and the dosage was gradually reduced according to the symptom relief of joint swelling and pain. In addition to MTX, patients in the SF + MTX group were administered 6.3 g of SF orally per day. The treatment continued for 3 months.

The primary outcomes included the symptoms of RA (swollen joint count (SJC), rheumatoid factor (RF), tender joint count (TJC) and visual analog score (VAS)) and the serum levels of osteoclast-related proteins (IL-6, IL-10, OPG and TRACP). The secondary outcomes included liver and kidney function indices.

RF, liver and kidney function indices were tested in the clinical laboratory before medication (at 0 months), 1 month and 3 months after medication. The levels of IL-6, IL-10, OPG, and TRACP in patient serum were measured using ELISA kits (MULTISCIENCES) according to the manufacturer’s protocol. Due to the COVID-19 epidemic, several patients missed their scheduled hospital visits for follow-up examinations.

### Data analysis

All data are presented as mean ± SEM. Intergroup comparisons were analyzed using one-way or two-way ANOVA. Tukey’s multiple comparison test was performed to evaluate differences between groups. Statistical significance was defined as *P* < 0.05.

## Results

### SF showed no obvious toxicity to ICR mice

**FIGURE 1:**
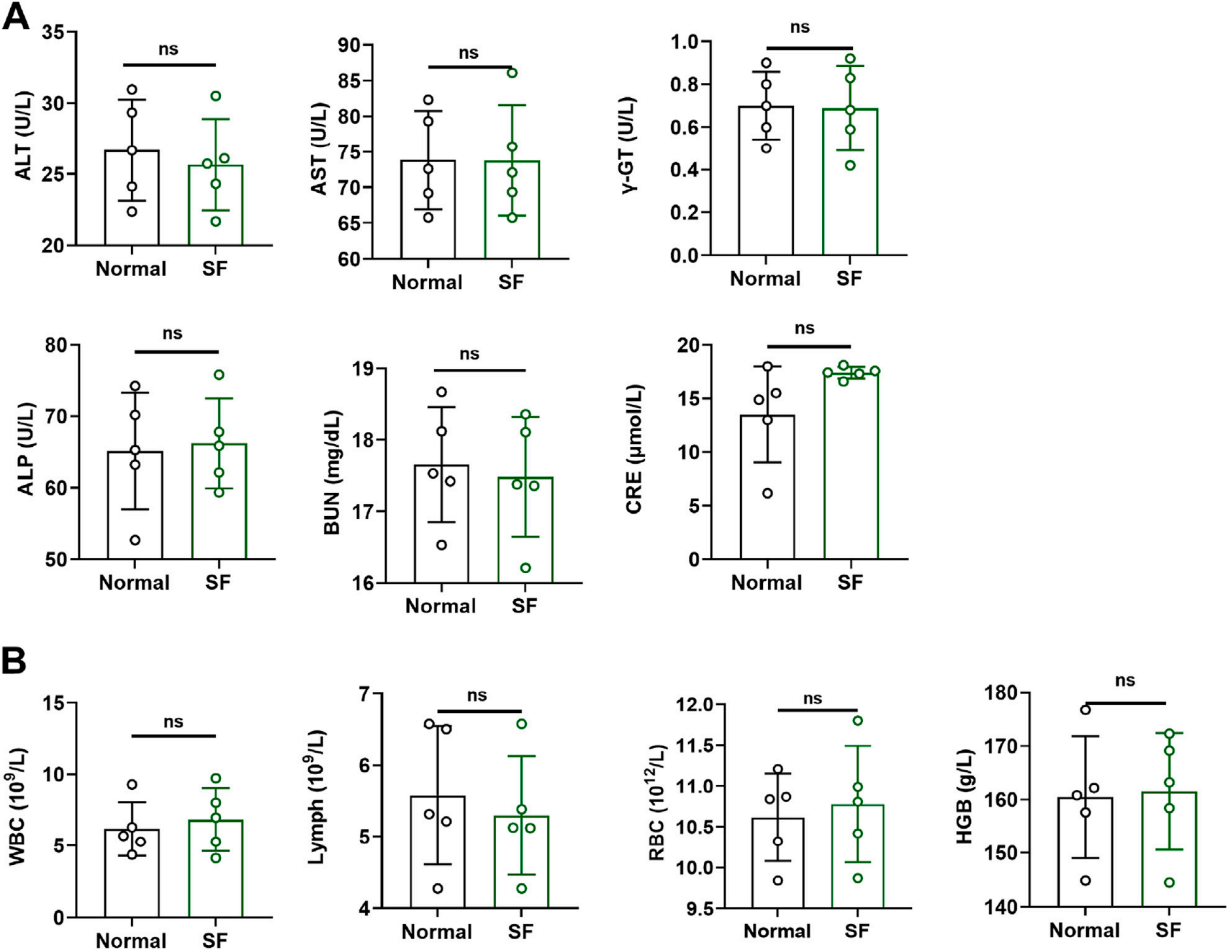
SF showed no obvious toxicity in ICR mice. **(A)** The serum levels of liver function indicators (ALT, AST, ALP and γ-GT) and kidney function indicators (BUN and CRE) in ICR mice (n = 5). **(B)** Biochemical tests of blood (WBC, Lymph, RBC and HGB) in ICR mice. ns: no significance, compared with the normal group (n = 5).

The SF prescription contains two animal-derived medicines and two plant-derived medicines. Toxicity evaluation in ICR mice revealed no abnormalities in hepatic/renal function parameters or hematological indices following 3 months of SF administration (Figures 1A,B), indicating that SF is safe and non-toxic in ICR mice.

### SF reduced the severity of arthritis, inhibited cartilage destruction and reduced the number of osteoclasts in CIA mice

**FIGURE 2:**
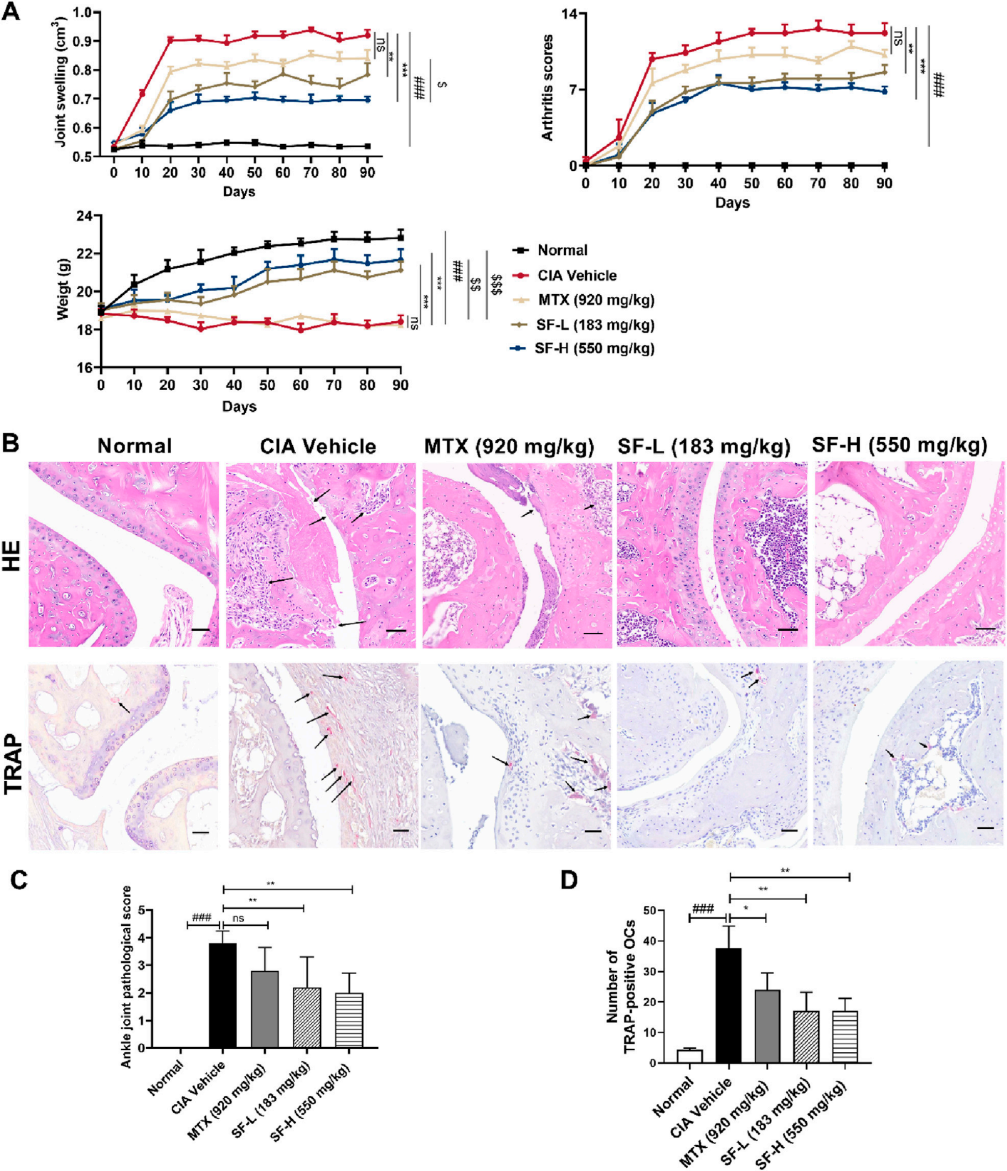
SF reduced the severity of symptoms in CIA mice. **(A)** Joint swelling, arthritis score of the paws, and weight of the mice in each group (n = 5). **(B)** Representative pathological images of ankle joints in each group after 90 days of treatment (scale bar = 100 μm). **(C)** Pathological scores of the ankle joints in each group (n = 5) after 90 days of treatment. **(D)** The number of osteoclasts characterized by TRAP staining in each group (n = 3). ns: no significance. *P < 0.05, **P < 0.01, ***P < 0.001, compared with the CIA vehicle group. ###*P* < 0.001, compared with the normal group. ^$^ *P* < 0.05, ^$$^ *P* < 0.01, ^$$$^ *P* < 0.001, compared with the MTX group.

Drug administration was initiated 28 days after primary immunization (designated as day 0 in Figure 2A). To assess the therapeutic effect of SF, we measured the hind paw joint swelling and arthritic scores in CIA mice. Compared with the CIA vehicle group, both the low-dose SF (183 mg/kg) and high-dose SF (550 mg/kg) groups exhibited significant reductions in the joint swelling from day 10, with arthritis scores showing significant improvement from day 20 through the end of administration (Figure 2A). No significant differences were observed between the MTX and CIA vehicle groups for the joint swelling and arthritis scores. Furthermore, both low and high doses of SF significantly attenuated weight loss in CIA mice compared to CIA vehicle group, whereas MTX group showed no significant protective effect (Figure 2A). SF was significantly more effective than MTX in improving joint swelling and body weight in CIA mice.

H&E staining suggested collagen-induced synovial hyperplasia and cartilage destruction in the ankle joints of the mice, while SF reversed these effects, and the pathological scores were much lower than those of the CIA vehicle group (Figure 2B). TRAP staining was used to identify osteoclasts in the ankle joints of the mice. A large number of osteoclasts appeared in the ankle joints of CIA mice, and the number decreased significantly after treatment with SF (Figure 2B).

### SF ameliorated bone erosion in the ankle joints of CIA mice

**FIGURE 3:**
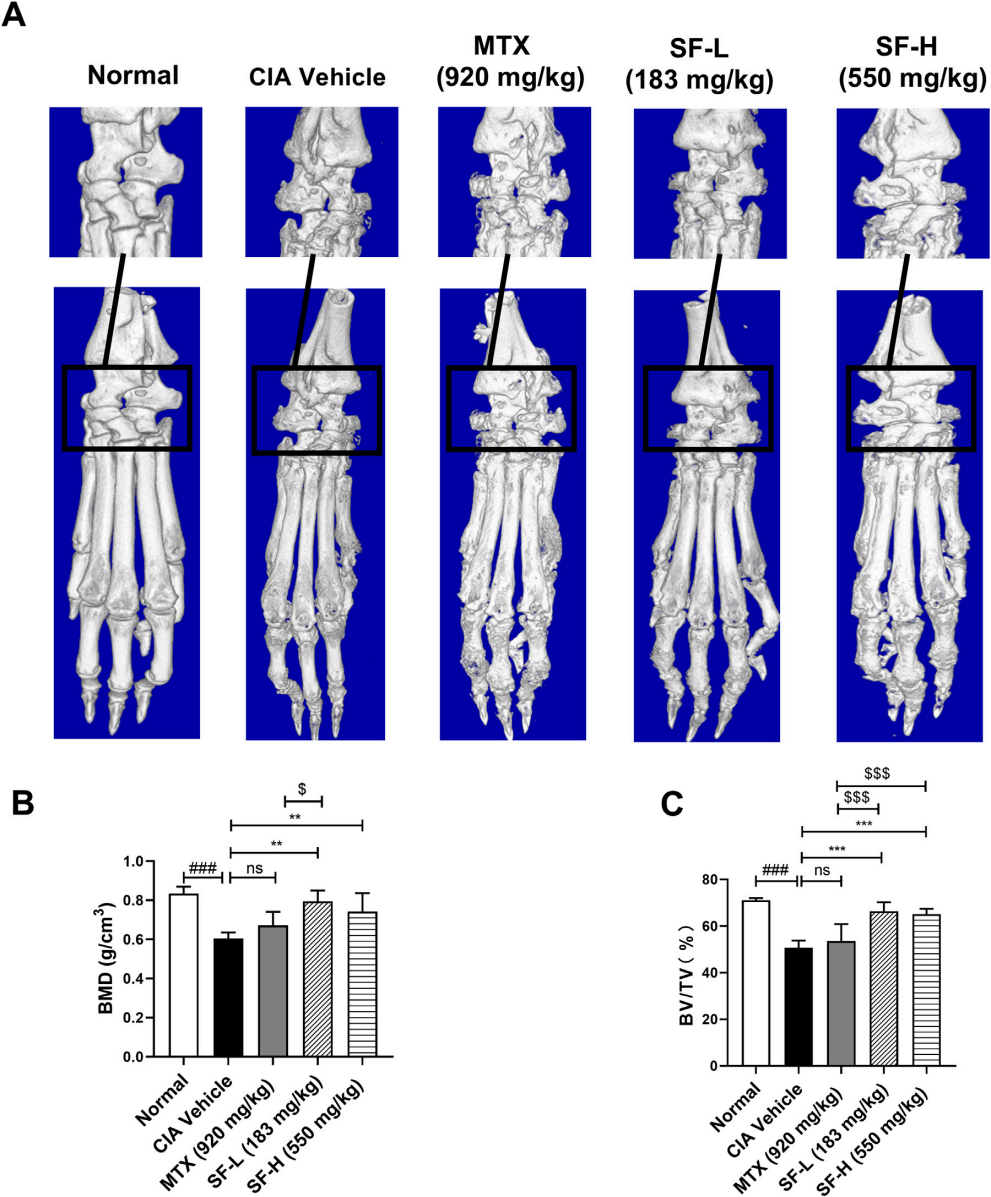
Amelioration of bone erosion in CIA mice after SF treatment. **(A)** Representative micro-CT images of the hind paws of the mice in each group. **(B)** Bone mineral density (BMD) and **(C)** bone fraction (BV/TV) of ankle joints in each group (n = 5). ns: no significance. **P < 0.01, ***P < 0.001, compared with the CIA vehicle group. ###*P* < 0.001, compared with the normal group. ^$^ *P* < 0.05, ^$$$^ *P* < 0.001, compared with the MTX group.

Micro-CT analysis was used to assess ankle joint bone erosion in CIA mice. In the normal group, the bone surface of the mice was smooth, and the bone structure was intact, whereas the CIA vehicle group exhibited severe bone erosion, joint bone destruction, and cavitation lesions (Figure 3A). Administration of both low- (183 mg/kg) and high- (550 mg/kg) dose SF significantly attenuated bone erosion in CIA mice (Figure 3A). Quantitative analysis revealed that bone mass parameters BMD and BV/TV were significantly reduced in the CIA vehicle group compared with controls. Notably, SF treatment markedly improved both BMD and BV/TV in CIA mice (Figure 3B). However, MTX treatment failed to significantly alter BMD or BV/TV compared with the vehicle, indicating that it had little effect on bone erosion.

### SF decreased the expression of osteoclast-related proteins in CIA mice

**FIGURE 4:**
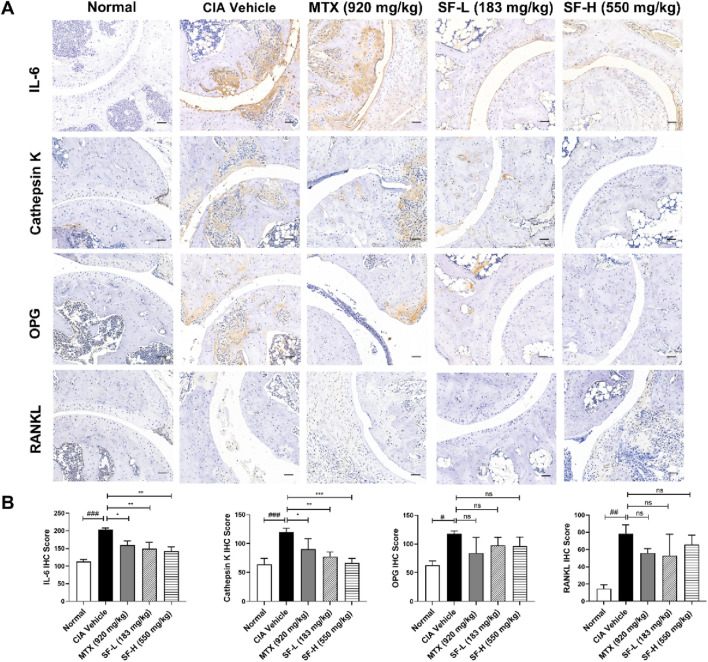
SF decreased the expression of osteoclast-related proteins in the ankle joints of CIA mice. **(A)** Representative immunohistochemical images of IL-6, cathepsin K, OPG and RANKL in the ankle joints of mice in each group (scale bar = 200 μm). **(B)** Quantification of IL-6, cathepsin K, OPG and RANKL in the ankle joints of mice in each group (n = 3). ns: no significance. *P < 0.05, **P < 0.01, ***P < 0.001, compared with the CIA vehicle group. #*P* < 0.05, ##*P* < 0.01, ###*P* < 0.001, compared with the normal group.

Immunohistochemical analysis revealed the expression levels of osteoclast-related proteins, including IL-6, cathepsin K, OPG and RANKL, in mouse ankle joints. The expression levels of these proteins were significantly higher in the CIA vehicle group than in the normal group (Figure 4). Both low and high doses of SF significantly reduced the expression levels of IL-6 and cathepsin K in the ankle joints but had no significant effect on OPG or RANKL expression in CIA mice. These findings suggest that SF might modulate the downstream signaling pathway of RANKL.

### SF suppressed osteoclast differentiation by inhibiting the RANKL/NF-κB signaling pathway

**FIGURE 5:**
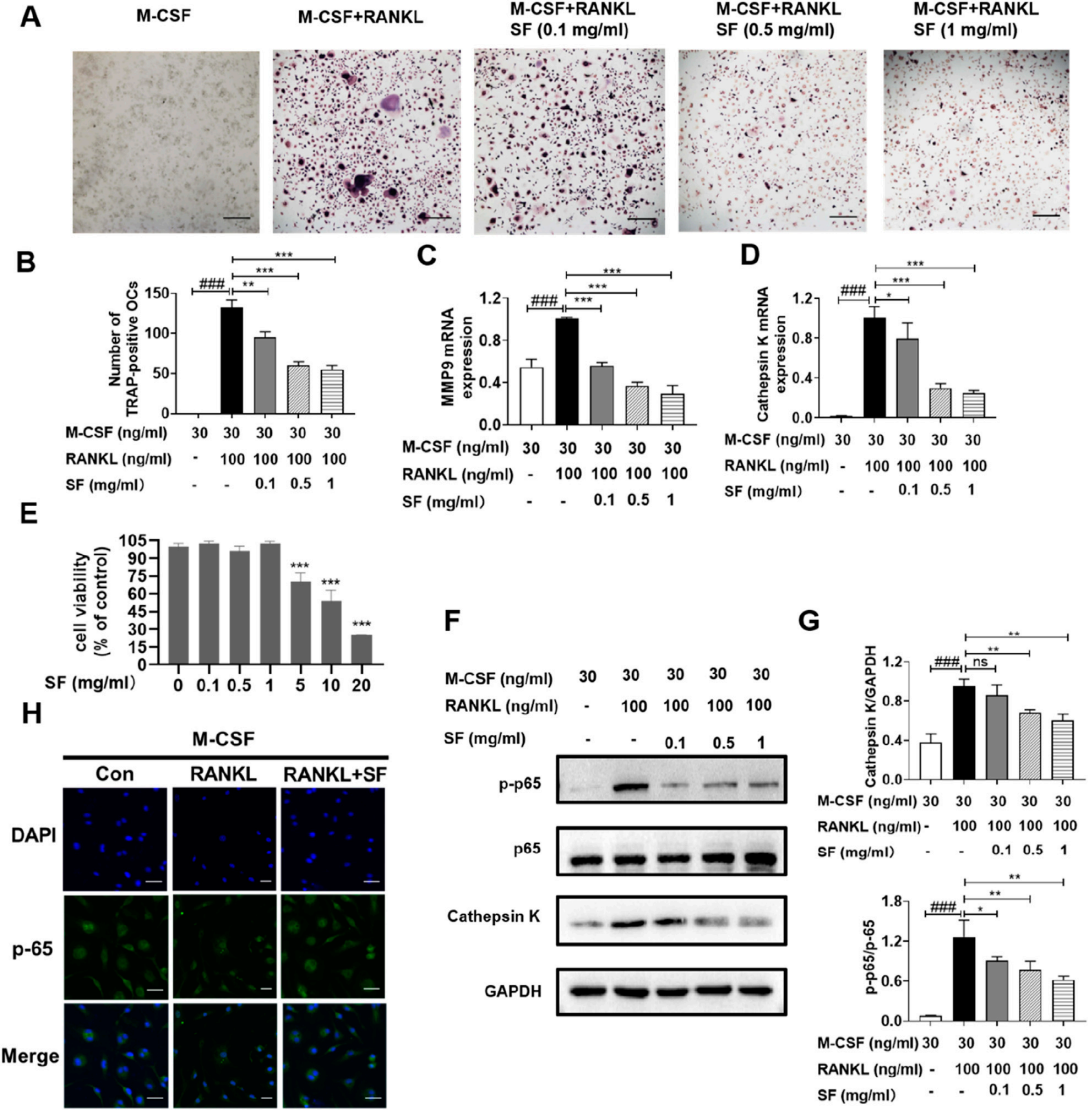
SF suppressed osteoclast differentiation by inhibiting the RANKL-stimulated NF-κB signaling pathway *in vitro*. **(A)** Representative images of TRAP-positive osteoclasts induced by RANKL after SF treatment (scale bar = 100 μm). **(B)** Quantification of TRAP-positive osteoclasts induced by RANKL after SF treatment (n = 3). **(C)** and **(D)** The mRNA expression levels of MMP9 and cathepsin K in bone marrow-derived mononuclear cells were determined by qPCR (n = 3). **(E)** Cell viability was evaluated using an MTT assay (n = 3). **(F)** Representative Western blotting images of p-p65, p65 and cathepsin K, with GAPDH serving as an internal reference. **(G)** Western blot analysis of p-p65/p65 and cathepsin K proteins (n = 3). **(H)** Representative immunofluorescence images of p65 nucleation in bone marrow-derived mononuclear cells (scale bar = 100 μm). ns: no significance. *P < 0.05, **P < 0.01, ***P < 0.001, compared with the M-CSF + RANKL group. ###*P* < 0.001, compared with the M-CSF group.

*In vivo* experiments demonstrated that SF reduced osteoclasts numbers, however, its effects on osteoclast differentiation and mechanism remained unclear. To investigate this, we examined SF’s inhibitory effects on osteoclastogenesis *in vitro*. Figures 5A,B shows that TRAP-positive cells (> three nuclei) were observed after stimulation with RANKL, and the number of TRAP-positive osteoclasts decreased in a dose-dependent manner after SF (0.1, 0.5, or 1 mg/mL) treatment. We next evaluated the effect of SF on the expression of the osteoclast-related genes cathepsin K and MMP9. Quantitative PCR analysis revealed that RANKL-stimulated bone marrow-derived mononuclear cells showed significantly elevated mRNA levels of cathepsin K and MMP9, which were markedly downregulated by 48-h SF treatment for (Figures 5C,D). The viability of bone marrow-derived mononuclear cells was not affected by SF at concentrations up to 5 mg/mL (Figure 4E).

The NF-κB signaling pathway is crucial for RANKL-stimulated osteoclast differentiation (Lin et al., 2017). Therefore, the effect of SF on the RANKL-stimulated NF-κB signaling pathway in bone marrow-derived mononuclear cells was further investigated. After RANKL stimulation, the protein expression level of p-p65 increased significantly but decreased after treatment with different concentrations (0.1, 0.5, or 1 mg/mL) of SF (Figures 5F,G). p65 translocated into the nuclei of bone marrow-derived mononuclear cells after RANKL induction, and SF blocked the nuclear translocation of p65 (Figure 5H). Consistently, the expression of the cathepsin K protein was also inhibited by 48 h of SF treatment (Figures 5F,G). These results indicated that SF might suppress osteoclast differentiation by inhibiting the RANKL-stimulated NF-κB signaling pathway.

### SF alleviated clinical symptoms and regulated the levels of bone erosion-related proteins in RA patients

**FIGURE 6:**
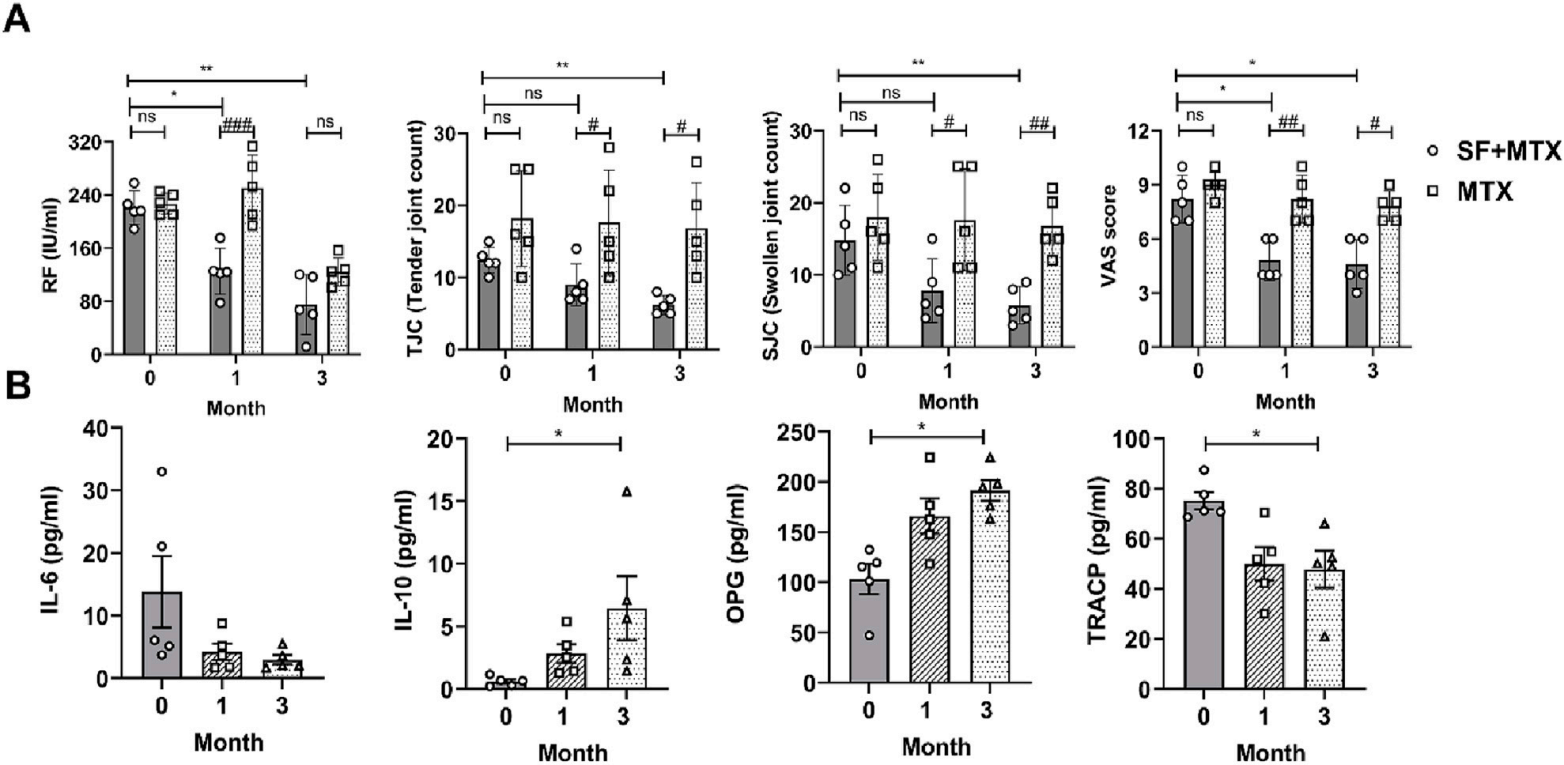
SF alleviated clinical symptoms and improved bone erosion-related protein levels in RA patients. **(A)** The serum rheumatoid factor (RF) concentration, tender joint count (TJC), swollen joint count (SJC) and visual analog scale (VAS) score of the RA patients in each group. **(B)** The serum levels of IL-6, IL-10, OPG and TRACP in the SF + MTX group. ns: no significance. #*P* < 0.05, ##*P* < 0.01, ###*P* < 0.001, comparison between the SF + MTX and MTX groups. *P < 0.05, **P < 0.01, compared with month 0.

In this randomized clinical trial, RA patients were allocated to an MTX group and an SF + MTX group at a 1:1 ratio for 3 months of continuous drug administration. With the prolongation of medication time, the RF of patients in the SF + MTX group decreased significantly (Figure 6A). Notably, the addition of SF effectively reduced the TJC, SJC and VAS scores of pain in RA patients, while MTX alone had no significant effect (Figure 6A).

SF not only ameliorated clinical symptoms but also improved the levels of bone erosion-related proteins in RA patients. After t the 3-month intervention, the inflammatory state of the RA patients in the SF + MTX group improved, the levels of the proinflammatory cytokine IL-6 decreased, and the levels of the anti-inflammatory cytokine IL-10 increased in the serum (Figure 6B). OPG is a decoy receptor of RANKL and can inhibit the activation and promote the apoptosis of osteoclasts. TRACP is an important marker of bone destruction and osteoclast activation. As shown in Figure 6B, the serum level of OPG increased, and that of TRACP decreased significantly after 3 months of SF treatment (Figure 6B).

### SF showed no obvious toxicity to the liver or kidney in RA patients

**FIGURE 7:**
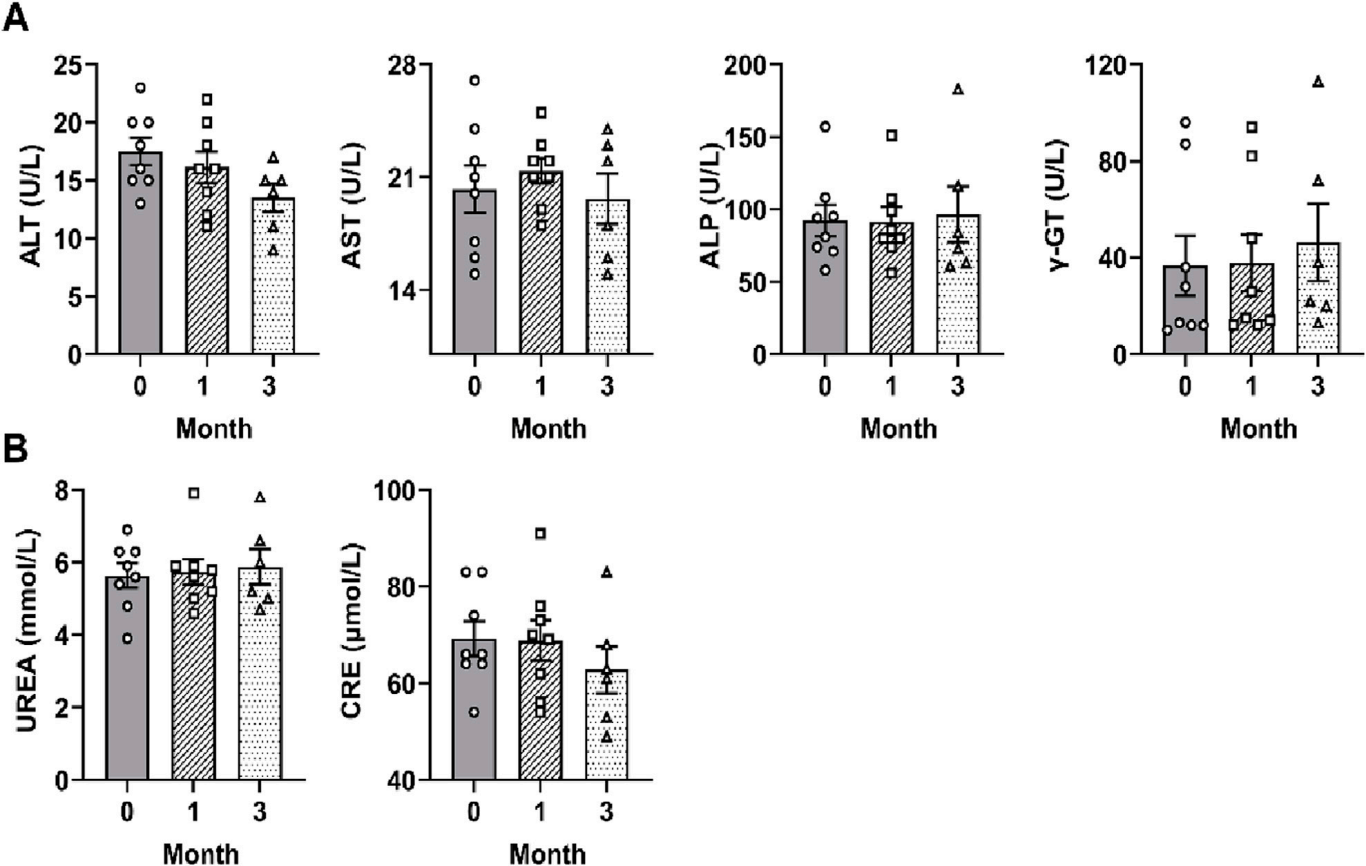
SF showed no obvious toxicity to the liver or kidney in RA patients. **(A)** The serum levels of liver function indicators (ALT, AST, ALP and γ-GT) in patients treated with SF + MTX. **(B)** The serum levels of kidney function indicators (BUN and CRE) in patients treated with SF + MTX.

To examine the safety of SF, the hepatic and renal function parameters of each patient were measured at one and 3 months after SF treatment. Hepatic function tests revealed that ALT, AST, ALP, and γ-GT levels remained within normal limits throughout the study period before and after SF treatment (Figure 7A). Similarly, renal function parameters BUN and CRE levels were maintained within normal ranges and showed no significant difference before and after SF treatment (Figure 7B). These findings demonstrate that SF has no obvious toxicity to the liver or kidney at the clinically used dose and is a safe and effective drug for treating RA.

## Discussion

Bone erosion-induced physical disability in RA patients is a serious problem, with a 43.5% disability rate observed within 5–10 years of disease progression (Brown et al., 2024; Meade et al., 2024). Current clinical therapeutics predominantly alleviate symptoms without effectively halting the progression of bone erosion in RA patients.

SF is an in-hospital preparation that has been used for the treatment of RA for over 5 years at the Affiliated Hospital of Integrated Traditional Chinese and Western Medicine, Nanjing University of Chinese Medicine. It is composed of astragali radix as a monarch medicine, scorpions and scolopendra as minister medicines, and black soybean seed coat as an adjunctive medicine. The main components of SF identified by UHPLC-QE-MS refer to our recent article and 716 compounds were obtained (Fan et al., 2025). According to “The Compendium of Materia Medica”, astragali radix possesses qi-replenishing properties, promotes musculoskeletal health, and enhances immune functions. Modern pharmacological studies have confirmed that astragali radix can alleviate symptoms of arthritis in rats through the OPG/RANKL/NF-κB pathway. Both scorpion and scolopendra have a long history of being used to relieve spasms and pain in China. The black soybean seed coats can activate blood circulation, reduce swelling, and detoxify.

Toxicity assessment demonstrated that SF exhibited no significant toxicity in ICR mice, indicating its safety and suitability for long-term treatment of RA. Notably, changes in composition and compatibility can affect the efficacy of traditional Chinese medicine. Our prior research revealed that SF (administered for 1 month) could delay the onset, alleviate the severity of arthritis, and restore Th17/Treg homeostasis by inhibiting Th17 cell differentiation in CIA mice (Hua et al., 2021). The present study investigated whether SF still has a therapeutic effect on bone erosion in patients with RA.

The CIA model is currently the gold standard model for *in vivo* RA investigation. Its pathological characteristics and autoimmune antigen mechanism are most similar to those of RA, with a success rate of 50%–70% and a morbidity rate of up to 100% (Schnabolk et al., 2021; Schuh et al., 2023). Consistent with our research group’s experience (Hua et al., 2021; Ling et al., 2021), the success rate of our CIA model is approximately 80%. After 3 months of administration, SF not only relieved RA symptoms but also reduced the number of osteoclasts and ameliorated bone erosion in the ankle joints of CIA mice. MTX, a first-line anti-RA drug used in the clinic, was used as a positive control agent in this study. Compared with MTX, SF was more effective at alleviating bone erosion and reversing the trend of weight loss in CIA mice.

In RA patients, excessive inflammatory cytokines release targets synovial cells, inducing pronounced synovial inflammation, hyperplasia and pain aggravation (Auréal et al., 2021; Cho et al., 2021; Sakthiswary et al., 2022). This inflammatory milieu promotes angiogenesis within the synovium, leading to pannus formation. The gradual expansion of the pannus causes major damage to the bone and cartilage within the joints, leading to the narrowing of the joint cavity, bone erosion, and, eventually, joint stiffness and calcification (Guo et al., 2018). Bone erosion is a core factor in the occurrence and progression of RA (Su et al., 2021). SF administration markedly attenuated synovial hyperplasia and inflammatory infiltration in ankle joints in CIA mice and reduced the destruction of articular cartilage. It significantly inhibited the development of bone erosion, improved joint deformation and cavitation, and increased the bone mass parameters in the ankle joints of CIA mice. Moreover, immunohistochemical analysis revealed that SF downregulated the expression of cathepsin K, a protein specific to osteoclasts, but had no significant effect on OPG or RANKL in the ankle joints of CIA mice. These findings suggest that SF may modulate the downstream signaling pathway mediated by RANKL.

Based on the *in vivo* results, we further verified SF’s inhibitory effect on bone erosion *in vitro*. SF significantly inhibited RANKL-induced osteoclastogenesis in bone marrow-derived mononuclear cells and downregulated the expression of the osteoclast-related genes cathepsin K and MMP9. The NF-κB signaling pathway downstream of RANKL is a key mediator of osteoclast differentiation (Novack, 2011). We subsequently investigated the molecular mechanism underlying SF’s inhibition of osteoclast differentiation through the RANKL/NF-κB signaling pathway was investigated. SF effectively suppressed the expression of p-p65 and prevented the nucleation of p65. These results suggest that SF might inhibit osteoclast differentiation by blocking the RANKL-mediated NF-κB signaling pathway.

Building upon the *in vivo* and *in vitro* experimental results, we conducted a preliminary clinical observation to evaluate SF’s affect on bone erosion in RA patients. Three-month SF adjunct therapy significantly alleviated the clinical symptoms, as evidenced by reducing the tenderness and swelling of the joints, improving the inflammatory state and reducing the serum RF level. Notably, the patients’ self-reported pain scores also decreased significantly after 1 month of medication and did not increase after 3 months. Existing evidence demonstrates that pro-inflammatory cytokine IL-6 stimulates osteoclastogenesis and accelerates bone erosion (Omokehinde and Johnson, 2020; Xiong et al., 2020; Loucks et al., 2023), whereas the anti-inflammatory cytokine IL-10 suppresses osteoclast fusion and activation (Geusens, 2012; Soliman et al., 2024). Our results revealed that SF treatment significantly modulated these cytokines, showing decreased IL-6 and increased IL-10.

Furthermore, we validated the clinical correlations between the serum OPG and TRACP levels and bone erosion. As a decoy receptor for RANKL, OPG competitively inhibits the binding of RANKL to RANK, thereby suppressing osteoclast differentiation and attenuating bone resorption (Stach et al., 2010). TRACP is mainly synthesized and secreted by osteoclasts, and its level and activity in serum can be used to predict the amount of osteoclasts and bone reabsorption (Schini et al., 2023). Our clinical data demonstrated that SF treatment significantly elevated OPG levels while reducing TRACP concentrations in RA patients. Importantly, longitudinal monitoring revealed no hepatic or renal function abnormalities following one- and three-month SF administration, confirming its favorable safety profile.

There are several limitations in this research. For example, the mechanism of bone erosion in RA was only preliminarily explored in osteoclasts, and more in-depth mechanisms as well as the active components of SF for RA treatment require further investigation. Limitations in the clinical research include the following: (1) the sample size of RA patients was small because of the COVID-19 pandemic; (2) the observation was not double-blinded; (3) bone erosion in RA patients was not detected by micro-CT; and (4) the key active components in SF responsible for bone erosion remain unclear. Future studies will address these limitations through the following approaches: 1) a randomized, double-blind and controlled clinical trial with a large sample of patients will be conducted to study the effect of SF on bone erosion in patients with RA, with multi-modal imaging to jointly detect bone destruction in patients; 2) implement LC-MS/MS-based untargeted metabolomics to systematically identify bioactive components in SF (particularly flavonoids and saponins) that modulate the RANKL/OPG signaling axis.

## Conclusion

This study demonstrates that SF is a safe traditional Chinese medicine formulation that effectively alleviates arthritis symptoms in RA patients. Our findings indicate that SF mitigates bone erosion by suppressing osteoclast differentiation through inhibition of the RANKL/NF-κB signaling pathway. The further development and application of SF have helped solve the problem that most clinically used RA drugs cannot inhibit the progression of bone destruction.

## Data Availability

The original contributions presented in the study are included in the article/supplementary material, further inquiries can be directed to the corresponding authors.

## Abbreviations

ALT, Alanine aminotransferase; ALP, Alkaline phosphatase; AST, Aspartate aminotransferase; BUN, Blood urea nitrogen; CIA, Collagen-induced arthritis; DMARDs, Disease-modifying anti-rheumatic drugs; ELISA, Enzyme-linked immunosorbent assay; GAPDH, glyceraldehyde-3-phosphate dehydrogenase; γ-GT, γ-glutamyl transpeptidase; H&E, Hematoxylin-eosin staining; HGB, Hemoglobin; IKK, Inhibitor of kappa B kinase; IL-6, Interleukin-6; IL-10, Interleukin-10; Lymph, lymphocyte; M-CSF, Macrophagecolony stimulating factor; MMP9, Matrix metalloprotein 9; MTX, Methotrexate; NF-κB, Nuclear factor kappa-B; NSAIDs, Nonsteroidal anti-inflammatory drugs; OC, Osteoclast; OPG, Osteoprotegerin; RA, Rheumatoid arthritis; RANK, Receptor activator of nuclear factor-κB; RANKL, Receptor activator of nuclear factor-κB ligand; RBC, Red blood cell, RT-CR: Real time-quantitative polymerase chain reaction; SF, Soufeng Sanjie formula; TRAP/TRACP, Tartrate-resistant acid phosphatase; WBC, White blood cell.
